# Sequence and structural analysis of the Asp-box motif and Asp-box beta-propellers; a widespread propeller-type characteristic of the Vps10 domain family and several glycoside hydrolase families

**DOI:** 10.1186/1472-6807-9-46

**Published:** 2009-07-13

**Authors:** Esben M Quistgaard, Søren S Thirup

**Affiliations:** 1 MIND Centre, Department of Molecular Biology, University of Aarhus, Gustav Wieds Vej 10C, DK 8000 Århus C, Denmark; 2 Department of Medical Biochemistry and Biophysics, Karolinska Institute, 17177 Stockholm, Sweden

## Abstract

**Background:**

The Asp-box is a short sequence and structure motif that folds as a well-defined β-hairpin. It is present in different folds, but occurs most prominently as repeats in β-propellers. Asp-box β-propellers are known to be characteristically irregular and to occur in many medically important proteins, most of which are glycosidase enzymes, but they are otherwise not well characterized and are only rarely treated as a distinct β-propeller family. We have analyzed the sequence, structure, function and occurrence of the Asp-box and s-Asp-box -a related shorter variant, and provide a comprehensive classification and computational analysis of the Asp-box β-propeller family.

**Results:**

We find that all conserved residues of the Asp-box support its structure, whereas the residues in variable positions are generally used for other purposes. The Asp-box clearly has a structural role in β-propellers and is highly unlikely to be involved in ligand binding. Sequence analysis of the Asp-box β-propeller family reveals it to be very widespread especially in bacteria and suggests a wide functional range. Disregarding the Asp-boxes, sequence conservation of the propeller blades is very low, but a distinct pattern of residues with specific properties have been identified. Interestingly, Asp-boxes are occasionally found very close to other propeller-associated repeats in extensive mixed-motif stretches, which strongly suggests the existence of a novel class of hybrid β-propellers. Structural analysis reveals that the top and bottom faces of Asp-box β-propellers have striking and consistently different loop properties; the bottom is structurally conserved whereas the top shows great structural variation. Interestingly, only the top face is used for functional purposes in known structures. A structural analysis of the 10-bladed β-propeller fold, which has so far only been observed in the Asp-box family, reveals that the inner strands of the blades are unusually far apart, which explains the surprisingly large diameter of the central tunnel of sortilin.

**Conclusion:**

We have provided new insight into the structure and function of the Asp-box motif and of Asp-box β-propellers, and expect that the classification and analysis presented here will prove helpful in interpreting future data on Asp-box proteins in general and on Asp-box β-propellers in particular.

## Background

The Asp-box is a phylogenetically ubiquitous sequence and structure motif. It was first described as a short repeat motif with consensus sequence S-X-D-X-G-X-T-W distinguishing bacterial from influenza sialidases or neuraminidases (the two terms are equivalent) [1]. For this reason it is also known as the bacterial neuraminidase repeat (BNR repeat). However, it is now clear that it is neither limited to sialidases nor to bacteria and we therefore prefer the term Asp-box, which is also the more prevalent term in the literature. The structure of *Salmonella typhimurium *LT2 sialidase [2] was the first structure to be determined of a bacterial sialidase or indeed any glycoside hydrolase family 33 (GH33) protein; bacterial, eukaryotic and some related viral sialidases all belong to this family. This structure revealed that the GH33 catalytic domain adopts a 6-bladed β-propeller fold with the Asp-boxes spanning the loop between the two outer strands, strand 3 and 4, of the four-stranded 'up-and-down' propeller blades [2], and that the Asp-box itself adopts a β-hairpin fold [2,3]. Structures of GH33 sialidases, trans-sialidases or sialidase-like proteins have since been determined from a variety of organisms including bacteria [2,4-9], trypanosomes [10,11], leech [12] and man [13]. Asp-box repeats have furthermore been observed in equivalent positions in the structures of several other β-propeller domains including the 6-bladed β-propeller of the bacteriophage K1F sialidase (GH58 family) [3,14], the tandem 7-bladed β-propellers of GH74 hydrolases [15-17] and the 10-bladed β-propellers of the Vps10-domain (Vps10-D) receptors, which stand out from all other structurally characterized Asp-box β-propellers by not being carbohydrate active enzymes [18,19]. The Asp-box is however not restricted to the β-propeller fold, but also occurs in the jelly-roll subrepeats of reelin [3,20], as singlet in the immuno-globulin-like (Ig-like) C-terminal domains of chitobiase (GH20 family) and sulfite oxidase and as singlet in the central 'up-and-down' β-sheet of the microbial ribonuclease fold [3,21]. Furthermore, a shorter variant of the Asp-box, missing the conserved glycine, has recently been identified. This motif occurs in the arabinase/levansucrase/invertase group of 5-bladed β-propellers and in some carbohydrate binding modules [15]. The Asp-box is just one among several repeats that are found in specific positions in β-propellers and defines a particular propeller family. Others include for example the WD40, kelch, YWTD (LDL receptor class B), PQQ (tryptophan docking motif), NHL and RCC1 repeats, which seem to generally function in folding and/or stabilization of the propellers. Such functions may also apply to the Asp-box [5,22], but functions in carbohydrate binding or secretion have also been proposed [2,3]. The Asp-box β-propeller family stands out from other β-propeller families in at least three ways; firstly Asp-box β-propellers are unusually irregular [23], secondly Asp-boxes are often missing in several and indeed sometimes most of the blades e.g. the 6-bladed β-propeller of bacteriophage K1F sialidase has just two Asp-boxes [14], and thirdly it is so far the only β-propeller family that encompass a 10-bladed fold. The considerable structural work carried out on Asp-box glycosidases reflects a fundamental interest in the mechanisms of these enzymes, but implications in diseases has also been a motivating factor. Bacterial sialidases serve parasitic nutritional functions for several pathogens and may act directly as virulence factors in some diseases e.g. cholera, gas gangrene, septiacemia, meningitis and cystic fibrosis [24], trypanosomal sialidases are crucial for the life-cycles of the parasitic species causing sleeping sickness and Chagas disease [25,26], human Neu3 sialidase is a potential target for cancer treatment [27], Neu1, Neu3 and Neu4 may find use in cancer diagnosis [28] and mutations in Neu1 are the cause of the lysosomal storage disease sialidosis [29]. Structural insight into the Vps10-D family has just recently been obtained by the structure determination of sortilin [19], but more structures will probably follow, since these proteins are now emerging as important players for central cellular functions in sorting and signaling and are firmly linked to several human disorders e.g. SorLA is implicated in Alzheimers disease, SorCS1 in type 2 diabetes and sortilin in age and trauma-induced neuronal cell death [30]. An examination of the structure and occurrence of the Asp-box as well as a discussion of its evolution and function has been published previously by Copley et al. [3]. However, at that time the only known Asp-box β-propellers were a number of sialdiases. Only very recently has it been realized, that Asp-boxes are also found in several β-propellers not belonging to the sialidase group [15]. Here we provide an updated and considerably more detailed analysis of the structure and function of the Asp-box motif and a much needed classification and comprehensive analysis of the Asp-box β-propeller family.

## Results and Discussion

### Sequence and structure of the Asp-box

**Figure 1:**
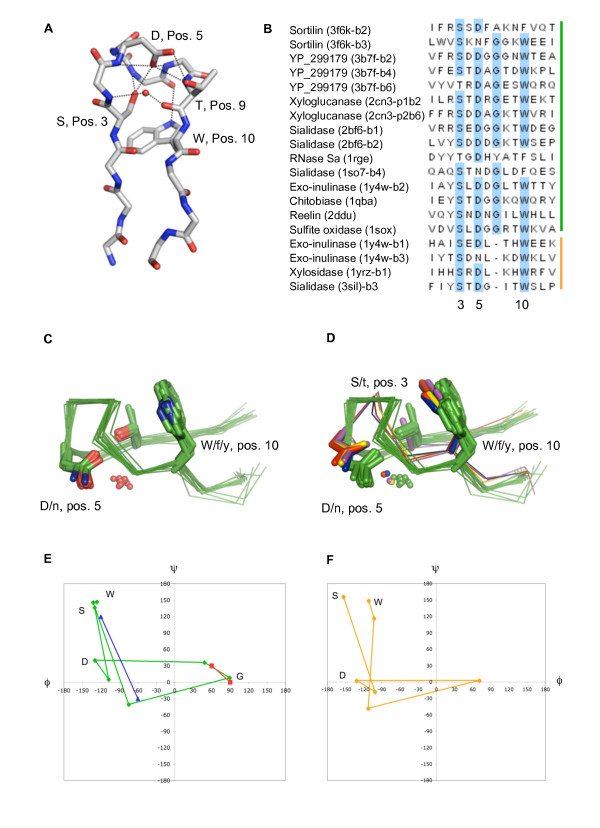
**Structure of the Asp-box and short-Asp-box motifs**. A. The main chain of the Asp-box of blade 1 of 2bf6 and side chains in highly conserved positions are shown as sticks. The structurally conserved water molecule is shown as a sphere. Coloration is by atom type. Hydrogen bonds involving the four shown side chains and the water molecule are represented by dotted black lines. B. Multiple structural alignment of fifteen Asp-boxes and four s-Asp-boxes. The sequences are labeled with protein name, pdb code, blade number (b) when extracted from β-propellers and propeller number (p) when extracted from a tandem β-propeller. The conserved S, D, G and W are shown in light blue. The vertical green and orange bars mark regular Asp-boxes and s-Asp-boxes respectively. C. Structural overlay of the Asp-boxes in (B). The main chain traces are shown as green ribbons, conserved side chains as sticks colored by atom type, and the conserved water molecules are shown as spheres. D. Same as in (C) but the short Asp-boxes are included. All Asp-boxes are uniformly green and the short-Asp-boxes are colored magenta, yellow, blue and red in order of appearance in the alignment in (B). E. Ramachandran plot for the residues SXDXGXXW of the Asp-box. The blue trace shows average values for phi and psi calculated for the 15 asp-boxes listed in (B). The positions of the conserved residues are indicated by their one letter abbreviation. The trace of standard type I' (red) and type VIII (blue) turns are shown for reference. F. Ramachandran plot for residues SXDXXXW of the s-asp-box. The orange trace shows average values of phi and psi calculated for the 4 s-asp-boxes listed in (B).

From the full Pfam BNR alignment, which is based on 5236 sequences, we find that the Asp-box consensus sequence can be expressed as X-X-(S/t)-X-(D/n)-X-G-X-(T/s)-(W/f/y)-X where capital letters represent the most conserved residue in a given position, small letters represent more or less commonly seen alternatives and X signifies variable positions. The additional variable residues at the beginning and end of the motif are included to indicate that they too are structurally conserved. Positions 3, 5, 7 and 10 are all strongly conserved (JalView conservation scores of 9–10 for the full Pfam BNR alignment), whereas the T/s of the 9^th ^position is only partly conserved (JalView score 4). The most preferred residues in positions 2, 6 and 8 are R, G and K respectively, but they were omitted from the expression due to very weak conservation (JalView score of 2). The Asp-box forms a structurally conserved hairpin loop, which is delineated by the two main chain hydrogen bonds between the residues at position 2 and 11, typically leaving the remaining residues to constitute a type 8:8 β-hairpin [31]. However, since the main chain carbonyl group at position 9 points into the loop towards the amides at positions 4 and 5 the loop can occasionally be classified differently (Figure 1A). The structural conservation is reflected in the distribution of phi and psi angles, and two standard β-turns [32] are easily recognized; a type I' turn at position 5–8 and a type VIII turn at position 7–10 of the motif (Figure 1E). The loop structure is further stabilized by an intricate network of hydrogen bonds involving the conserved residues of the Asp-box motif and a water molecule in a conserved position (Figure 1A). The water molecule is coordinated by the main chain amide in the 4^th ^and the main chain carbonyl in the 9^th ^position and most often also the side chain hydroxyl in position 9 when a threonine or serine is found here (although not in the example shown in Figure 1A). The hydrogen bonding network, though slightly variable, is in general constituted by the following interactions; the conserved hydroxyl group at position 3 forms a hydrogen bond to the main chain amide at position 6, the conserved D/n is within hydrogen bonding distance of several main chain amides and also hydrogen bonds to the side chain hydroxyl of the S/t in position 3 as well as T/s if present in position 9. The Cβ of S/t in position 3 forms Van der Waal contacts to the side chain of the conserved aromatic residue in position 10 and when this is conserved as a tryptophan, which is strongly favored, a hydrogen bond is generally formed between the Nξ1 atom and the main chain carbonyl of the glycine in position 7. The conserved residues thus clearly contribute to maintaining the fold. Divergence from the consensus sequence is tolerated to some extent, but no other residues than D/n are found in position 5 and no non-aromatic residues are found in position 10 in any known structures. Furthermore, rare examples are found of Asp-boxes that conform to the consensus sequence but are distorted in structure. Two such examples are the Asp-box in blade 5 of *M. viridifaciens *sialidase [5] and the Asp-box in blade 6 of sortilin. In the case of sortilin, the conserved aspartate in position 5 is forced into an unusual position by forming ionic interactions with an arginine side chain of the interacting 10cc-b domain, which leads to a marked change in the loop structure. A motif that appears to be derived from the Asp-box has recently been identified. It lacks the conserved G and so conforms to the simplified consensus S/t-X-D/n-X-X-X-W/f/y [15] (Figure 1B). The structures of these motifs, which we name s-Asp-boxes, s is for short, are conserved and overall similar to that of the regular Asp-box in positioning of the conserved side chains and the structurally conserved water molecule (Figure 1D). The absence of the glycine in position 7 is compensated for by changes in the psi-angle of the two preceding residues and in the phi angle of position 8. As a result of this, no standard β-turns are recognized in the s-Asp-box, though the traces of the two loops in the Ramachandran plot are similar (Figure 1E–F). Due to the shorter loop, the hairpin of the s-Asp-box is typically classified as a type 7:7 β-hairpin.

### Occurrence of Asp-boxes in different folds

We have performed a structure-based search for the Asp-box motif in the pdb database and find that it in addition to the already known occurrences listed in the introduction, also occurs as repeats in the 7-bladed β-propeller of the functionally uncharacterized bacterial YP_299179.1 protein labeled 'glycosyl hydrolase' in the NCBI database, as singlet in the 5-bladed β-propeller of inulinase (GH32 family) and not surprisingly as singlet in the Ig-like dimerization domains of bacterial sulfite dehydrogenase and nitrate reductase, both of which are related to the dimerization domain of eukaryotic sulfite oxidase, which is already known to contain a single Asp-box [3]. We conclude that Asp-boxes occur in 5-bladed (rare occurrence in GH32), 6-bladed (GH33, GH58), 7-bladed (GH74, 'YP_299179.1 family') and 10-bladed (Vps10-D) β-propellers, in the β-sandwich folds of sulfite oxidase, some structurally related enzymes, in reelin and in chitobiase (GH20 family), and finally in the microbial ribonuclease domain characteristic of barnase, binase, RNase Sa, Sa2 and Sa3. A structure-based search has also been performed for the s-Asp-box variant. This motif is found as repeats or singlet in the 5-bladed β-propellers of the GH32 and GH43 families and as singlet in the β-sandwich fold of many family 32 carbohydrate binding modules (CMB32 domains), which is in good agreement with previous observations [15]. In addition it occurs in the F5/F8 type C domain of neuropilins and coagulation factors V and VIII, which is not surprising, since these domains adopt the same fold as the CBM32 domain. Finally it occurs in the central β-sandwich of the PqqB coenzyme PQQ synthesis protein and in the anti-parallel β-sheet of the N-terminal domain of type III pantothenate kinase (type III PanK) from *Thermotoga maritima*. The fold of the latter belongs to the widespread Ribonuclease H-like family [33], which does not generally encompass an s-Asp-box. Indeed an s-Asp-box is even missing in type III PanK from *Bacillus anthracis *and it therefore seems likely that it has evolved relatively recent in *Thermotoga maritima *by chance convergence.

### Structural contexts and functions of Asp-boxes in non-propeller folds

**Figure 2:**
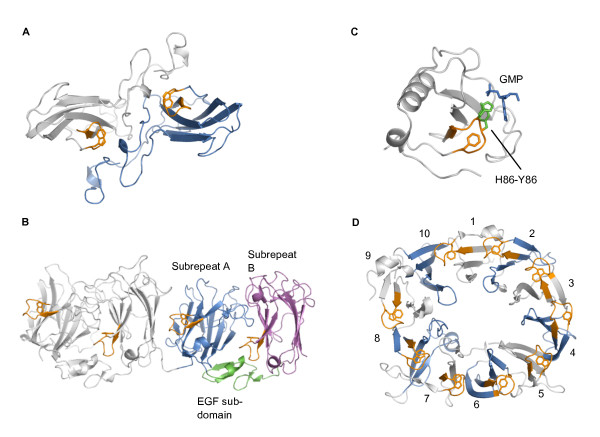
**Representative Asp-box proteins**. A. The dimer of the sulfite oxidase (1sox) dimerization domain shown in cartoon representation with the individual monomers colored grey and blue respectively and with the Asp-boxes colored orange. The conserved aromatic residues in the 10^th ^position of the Asp-boxes are shown in sticks. B. Two consecutive reelin repeats (2e26). The first repeat is colored grey, and the second is colored by sub-domain composition; subrepeat A is blue, the EGF subdomain is green and subrepeat B is magenta. The Asp-boxes are depicted as in (A). C. RNase Sa in complex with GMP (1gmp). The protein is grey, GMP is in blue sticks and the Asp-box is depicted as in (A) except that the side chains of H85-Y86 are shown in green sticks. D. The Asp-box β-propeller domain of sortilin (3f6k). The blades are numbered, uneven blades as well as the loops between blades are colored grey and even numbered blades are blue. The Asp-boxes are depicted as in (A).

In Ig-like and jelly-roll β-sandwich domains the Asp-box is rather surface exposed and is often involved in supporting domain-domain interactions. The Asp-box bends away from the sandwich interface and the conserved aromatic residue in position 10 is found on the outer face of one of the two β-sheets where it interacts quite extensively with several other side chains belonging to the same sheet. A C-terminal Ig-like domain with a single Asp-box is found in both sulphite oxidase and chitobiase. In sulfite oxidase the Asp-box contributes to the homodimer interaction face (Figure 2A), but chitobiase is a monomer and it is not clear if the Asp-box plays any functional role for this protein, although it may be noted that it is involved in crystal packing of 1qba. Reelin is a large protein containing several so called reelin repeats, which are compact domains consisting of three modules; an EGF sub-domain and two β-sandwich or jelly-roll sub-domains called subrepeats A and B, which both contains a single Asp-box (Figure 2B). As has been reported previously, the Asp-box in subrepeat B seems to ensure that the three individual modules form a single compact entity by binding to subrepeat A of the same repeat [20]. We find however, that the Asp-box in subrepeat A also seems to support structural integrity. It contributes to the interface between two reelin repeats by interacting with subrepeat B of the preceding repeat and may thus support the rodlike super-structure of the protein. In bacterial ribonucleases the single Asp-box is found in a surface exposed position in the central β-sheet (Figure 2C) and has several functions; H85 and Y86 of RNase Sa representing positions 6 and 7 of the Asp-box are involved in binding of the nucleotide [34] and the histidine in position 6 is also directly involved in catalysis [35]. In addition the tyrosines in Asp-box positions 1, 2 and 7 of RNase Sa (Y80, Y81, Y86) form hydrogen bonds, that have been shown to significantly influence the stability of the protein [36].

### Structural contexts and functions of Asp-boxes in β-propellers

In β-propellers Asp-boxes are invariantly found in the loops between strand 3 and 4 of the sheets/blades and are rather surface exposed (Figure 2D). They always form contacts to the previous blade or at least to the loop connecting the blade to the previous blade and additional stabilizing contacts are often formed to the succeeding blade as well. The distance between blades and the actual interactions formed are quite variable, excepting that the conserved aromatic residue in position 10 almost always forms van der Waals interactions to the preceding blade and/or to the loop connecting the blade to the preceding blade. Further common interactions are from positions 6 and 8 to the preceding blade, from position 1 to the loop connecting the blade to the preceding blade and from positions 2 and more rarely 4 to the succeeding blade. It has previously been proposed that the PQQ repeat/tryptophan docking motif is related to the Asp-box since this motif also has a conserved aromatic residue in the beginning of strand 4, which is likewise involved in blade to blade interactions [5]. We find however that the loop between strand 3 and 4 is not overall similar in the two motifs, and furthermore the conserved aromatic residue of the PQQ motif points in the opposite direction and thus interacts with the succeeding rather than the preceding blade.

Nonetheless we can conclude that the repeats of Asp-box β-propellers appear to have a structural function in mediating blade to blade interactions reminiscent of other propeller-associated repeats. This notion is furthermore in agreement with mutational analysis carried out on the *Clostridium perifringens *NanH sialidase, since mutating conserved Asp-box positions in this enzyme resulted in delayed or abolished secretion and reduced enzymatic activity indicative of misfolding [22].

Furthermore, the W240R mutation in position 10 of a conserved Asp-box in human Neu1 sialidase causes type II sialidosis [37], and although this was hypothesized to be caused by altered surface properties, destabilization or misfolding of the enzyme seems to be valid alternative explanations. However, the fact that Asp-boxes are often missing in several blades of Asp-box β-propellers, may suggest that they are not generally of paramount importance for dictating or stabilizing the fold and indeed additional or alternative functions in carbohydrate binding or as secretion signals have been proposed [2,3]. The notion that Asp-boxes may bind carbohydrate stems from the observations that they mostly occur in carbohydrate active enzymes and form polar surface exposed loops [3], but it should also be mentioned that the two most conserved residues, D and W, are generally highly favored in carbohydrate binding sites [38-40]. Asp-boxes have however never been observed to bind carbohydrate even though a great many known structures of Asp-box proteins have been determined from crystals grown or soaked with carbohydrates. In addition, the conserved D and W side chains are almost completely unavailable for interacting with carbohydrate, since the aromatic residue is buried between two blades and the aspartate is secluded by internal interactions of the Asp-box. Furthermore, a specific function in binding carbohydrate enzyme substrates is made unlikely by the facts that Asp-boxes are found on the opposite face of the active site of glycosidase Asp-box β-propellers, that the carbohydrate binding sites of the occasionally found flanking carbohydrate binding modules [41,42] are likewise far away from the Asp-boxes, and that Asp-boxes are present in non-carbohydrate active proteins such as the Vps10-D receptors. A general function in cell adhesion by binding to cell-surface glycoconjugates is likewise unlikely, since such a function is in poor agreement with the existence of cytosolic GH33 proteins and since the Asp-boxes of sortilin are found on the same propeller face as two bulky N-linked glycosylations. Asp-boxes may however affect secretion by way of their polar properties and if indeed the motif facilitates folding, this function will likely translate into an effect on the rate of secretion as well. There is however not much evidence to suggest that Asp-boxes serve as actual signal sequences for secretion. Indeed the existence of cytosolic GH33 proteins makes it rather improbable that the primary function of Asp-boxes is to aid in secretion, as has also been pointed out previously [3]. The s-Asp-box motif is, like the Asp-box, always found in the loop between the third and fourth strand of a subset of propeller blades when occurring in β-propellers, but it is usually found in just one or two blades. In the CBM32/F5/F8 type C domain it is invariantly found in a specific loop connecting two outer strands of the β-sandwich. Notably, this loop is found directly opposite of the CBM32 carbohydrate binding site and there is therefore no reason to believe that it can function in carbohydrate binding. If any, it probably has a structural function similar to that of the Asp-box.

### Phylogenetic distribution and diversity of Asp-box β-propellers

Asp-box β-propellers have so far been described from most major branches of life as well as from some viruses and bacteriophages. To further investigate the phylogenetic distribution, we have performed an Asp-box InterPro search. InterPro 18.0 covers 75.6% of UniProtKB and almost exclusively detects proteins with at least two Asp-box repeats, thus only 11 out of 1311 hits in bacteria and 2 out of 562 hits in eucaryotes were found to be single Asp-box proteins. The far majority are thus putative Asp-box β-propellers, although it should be mentioned that reelin and possibly a few other non-propeller proteins also contain more than one Asp-box. This search revealed that putative Asp-box β-propellers are indeed present in all major branches i.e. bacteria, archea, protozoans, metazoans, plants and fungi although with some curious absences in the metazoan branch. It has been noted previously that no Vps10-D proteins are present in the *Drosophila *genus or the *Nematoda *phylum although this protein family is generally widespread in animals [30]. We now find that these puzzling absences actually apply to all Asp-box repeat proteins. Most putative Asp-box β-propellers are found in bacteria. Indeed the Asp-box family appears to be one of the most common β-propeller families in bacteria together with the PQQ and WD40-related repeat families (Table 1). On the other hand, the number of kelch and especially WD40 repeat proteins far exceeds that of Asp-box repeat proteins in eukaryotes. The functional range of the so far characterized Asp-box β-propellers is limited, but we find that proteins with two or more Asp-boxes can co-occur with at least 70 different domains and motifs, implying that Asp-box proteins are much more variable and diverse than can be appreciated from the currently available experimental data. Most of these domains are implicated in carbohydrate binding, cell surface adhesion, protein-protein interaction or different kinds of hydrolysis, or have structural or unknown functions (see additional file 1: Suppl_Box1.pdf for list of domains)

**Table 1:** Phylogenetic distribution of Asp-boxes and other propeller-associated repeats

	Viruses	Archea	Bacteria	Eucaryota
Asp-box (BNR)	20	9	1300	560
WD40 repeat-like	9	8	913	17786
Kelch repeat type 1 and type 2	183	10	416	3067
PQQ repeat	0	120	1378	254
YWTD repeat	0	7	30	428
YVTN beta-propeller repeat	0	50	347	0
NHL repeat	0	76	741	379
RCC1	8	9	259	1128

The phylogenetic distributions of proteins that contain Asp-box repeats (at least two) or repeats belonging to one of seven other major propeller-associated repeat families.

### Overview and classification of known Asp-box β-propeller structures

**Figure 3:**
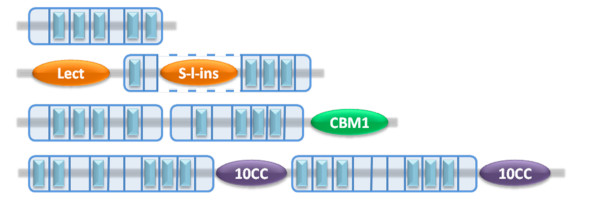
**Organizational types of Asp-box β-propeller proteins**. The four organizational types identified from known structures are represented by specific examples. The Asp-boxes are shown as light blue rectangles, Asp-box propeller boundaries are framed in blue, known or predicted blade boundaries are marked by vertical blue lines inside the frame and other domains found in the selected examples are shown as labeled ovals. Type 1 (top); a stretch of Asp-boxes that is not interrupted by any domains forms one β-propeller, shown here is YP299179.1 (3b7f). Type 2 (second from top); a stretch of Asp-boxes is interrupted by an inserted domain, but yet forms a single propeller, shown here is *Vibrio Cholera *sialidase (1w0p). 'Lect' is a lectin-like domain and 'S-l-ins' represents Pfam: Sial-lect-inser, which is a lectin binding protein-protein interaction domain, which has a fold similar to that of 'Lect'. Type 3 (second from bottom); a stretch of Asp-boxes not interrupted by any domains forms two tandem propellers, shown here is the GH74 protein Avicelase III from *Aspergillus aculeatus *(BAA29031.1). 'CBM1' stands for carbohydrate binding module 1. Type 4 (bottom); an interrupted stretch of Asp-boxes forms two propellers, shown here is Vps10p from yeast (AAA18831.1). '10CC' represents the two neighboring 10CC-a and 10CC-b domains.

An overview of all known structures of Asp-box β-propellers and their basic characteristics is given in Table 2. It should be pointed out however, that the many hundreds of uncharacterized proteins with Asp-box repeats (Table 1), suggests that several additional families remain to be discovered. The so far known structures of Asp-box β-propellers belong to the GH33, GH58 and GH74 glycoside hydrolase families, an uncharacterized family defined by YP299179.1 and the Vps10-D family. It is noticeable that there is a considerable variation in the number of Asp-boxes and that no known n-bladed structure encompasses n Asp-boxes. In all known Asp-box β-propellers, except YP299179.1, the N-terminal strand of the propeller domain replaces strand 4 of the last blade in a so called 1+3 clamping or "Velcro" closure arrangement as in most other β-propellers [43]. The maximum number, n-1, of asp-boxes is reached in both sortilin and *M. viridifaciens *sialidase, and most Asp-box β-propellers are just one or two Asp-boxes short of a full complement. In the cases where Asp-boxes are missing in one or more blades, it is quite variable in which blades the absences occur, even within the same sequence families. This is in good agreement with a general function in stabilization, since such a function would be equally important in any blade due to the inherent symmetry of the β-propeller fold. The s-Asp-box motif is found in the 5-bladed folds of the GH32 and GH43 families, but usually occurs in only one or two copies and is even missing entirely in some GH43 β-propellers (not shown in Table 2). Notably there are single examples of proteins where an s-Asp-box is found in an Asp-box β-propeller (3sil) or vice versa (1y4w). This, together with the structural similarities and equivalent positions of the two motifs, strongly suggests that all or most Asp-box and s-Asp-box β-propellers share a common ancestor. This is furthermore in agreement with recent results from a systematic pair wise analysis of the hidden Markov model profiles of sixty representative β-propellers, showing that the eleven Asp-box and s-Asp-box β-propellers included in the study were more similar to each other than to other β-propellers [15]. As an alternative to sequence-based classification, Asp-box β-propellers can also be classified by the number of blades i.e. 6-, 7- or 10-bladed β-propellers, or by how the Asp-box motifs are organized with respect to other domains and motifs in the proteins (Figure 3). Both of these classification schemes are useful for illustrating the structural plasticity of Asp-box β-propellers and may also be valuable for future structure predictions. Based on the currently known structures we find that Asp-box β-propellers can be divided into at least four different organizational types. In type 1 a continuous stretch of Asp-box repeat blades is associated with a single propeller domain. This is the simplest and most prevalent type and includes most sialidases, YP_299179.1 and the vertebrate Vps10-D receptors. Type 2 also forms just one propeller domain, but here the blades are interrupted by an inserted domain as in *V. cholera *sialidase. In type 3, which encompass the GH74 proteins, two propellers are formed from a continuous stretch of Asp-box repeat blades equivalent to two type 1 propellers in immediate succession. Finally, in type 4 two propellers are formed from a stretch of Asp-boxes that is interrupted by one or more domains, as can be deduced to apply to yeast Vps10p. Note that these different types may be difficult to distinguish from sequence data alone, which can make it quite challenging to perform theoretical modeling of Asp-box β-propellers of structurally uncharacterized families.

**Table 2:** Overview of all known structures of β-propellers containing Asp-box or s-Asp-box motifs

Family	Protein	Species	# Blades	# Asp- boxes	Blades with Asp-box	# short- Asp- boxes	Blades with short-Asp-box	# pdb files	Highest resolution pdb file
GH33	Sialidase NanI	*Clostridium perfringens*	6	4	1, 2, 3, 4	0	-	4	2bf6 (0.97 A)

	Sialidase	*Micromonospora viridifaciens*	6	5	1, 2, 3, 4, 5	0	-	10	1w8o (1.70 A)

	Sialidase	*Salmonella typhimurium*	6	3	1, 2, 4	1	3	5	3sil (1.05 A)

	Sialidase NanA	*Streptococcus pneumoniae Hungary19A-6*	6	4	1, 2, 3, 4	0	-	1	2vvz (2.50 A)

	Sialidase NanB	*Streptococcus pneumoniae TIGR4*	6	4	1, 2, 3, 4	0	-	3	2vw2 (1.70 A)

	Sialidase	*Vibrio cholera*	6	4	1, 3, 4, 5	0	-	3	1w0p (1.60 A)

	Cytoplasmic sialidase Neu2	Homo sapiens*	6	3	2, 3, 4	0	-	14	1so7 (1.49 A)

	Intramolecular trans-Sialidase	Macrobdella decora*	6	4	1, 2, 3, 4	0	-	5	2sli (1.80 A)

	Trans-sialidase	Trypanosoma cruzi*	6	3	1, 3, 4	0	-	13	1ms9 (1.58 A)

	Sialidase	Trypanosoma rangeli*	6	3	1, 3, 4	0	-	9	1n1t (1.60 A)

	Pseudaminidase	*Pseudomonase aeruginoasa*	6	3	2, 3, 4	0	-	1	2w38 (1.90 A)

GH58	Endosialidase	*Bacteriophage K1F †*	6	2	1, 4	0	-	2	1v0e (1.90 A)

GH74	Xyloglucanase	*Clostridium thermocellum*	7 + 7	4 + 5	2, 3, 4, 6/2, 3, 4, 5, 6	0	-	2	2cn3 (1.95 A)

	Cellobiohydrolase	Geotrichum sp. M128*	7 + 7	4 + 5	2, 3, 4, 6/2, 3, 4, 5, 6	0	-	2	1sqj (2.20 A)

Not known	YP_299179.1	*Ralstonia eutropha*	7	5	2, 3, 4,	0	-	1	3b7f (2.20 A)

Vps10 domain	Sortilin	Homo sapiens*	10	9	1, 2, 3, 4, 5, 6, 7, 8, 9	0	-	1	3f6k (2.00 A)

GH32	Exo-inulinase	Aspergillus niger*	5	1	2	2	1, 3	3	1y4w (1.55 A)

	Invertase	*Thermotoga maritima*	5	0	-	3	1, 2, 3	2	1w2t (1.87 A)

	Cell-wall invertase	Arabidopsis thaliana*	5	0	-	2	1, 3	5	2ac1 (2.15 A)

	Fructosidase	*Cichorium intybus*	5	0	-	2	1, 3	5	1st8 (2.35 A)

GH43	Xylosidase	*Bacillus halodurans*	5	0	-	1	1	1	1yrz (2.00 A)

	Xylosidase	*Bacillus subtilis*	5	0	-	1	1	1	1yif (1.80 A)

	Arabinase	*Bacillus subtilis*	5	0	-	1	1	1	1uv4 (1.50 A)

	Arabinase	*Cellvibrio japonicus*	5	0	-	1	1	3	1gyh (1.89 A)

	Xylosidase	*Clostridium acetobutylicum*	5	0	-	1	1	2	1y7b (1.60 A)

	Xylosidase	*Geobacillus stearothermophilus*	5	0	-	1	1	4	2exh (1.88 A)

	Arabinase	*Geobacillus thermodenitrificans*	5	0	-	1	1	1	1wl7 (1.90 A)

	Xylosidase/ara- binofuranosidase	*Selenomonas ruminantium*	5	0	-	1	1	1	3c2u (1.30 A)

There are currently 76 known structures with Asp-box β-propeller domains. These have 2–9 Asp-boxes and represent 16 different proteins and 5 different protein families. There are furthermore 29 known structures with 1–3 s-Asp-box motifs, which represent 12 different proteins and 2 families of 5-bladed β-propellers. Most known structures are from bacteria (unmarked), but there are also several from eukaryotes (species marked by *) and one from a bacteriophage (marked by †).

### Putative hybrid β-propellers

**Figure 4:**
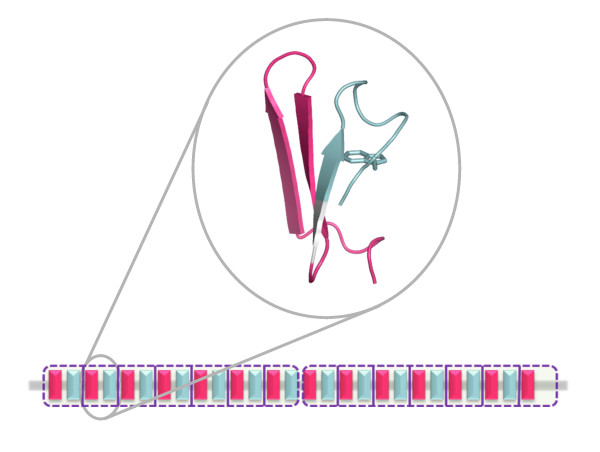
**Putative hybrid β-propeller**. The YP_001558799.1 hypothetical protein from *Clostridium phytofermentans *(Interpro accession A9KRI6) is an example of a putative Asp-box/Reg_prop hybrid β-propeller. A single blade (blade 2) of a fold recognition model is shown along with a schematic of the sequence. The putative hybrid propeller boundaries suggested by fold recognition are marked by stippled purple frames and the likely boundaries of the blades are indicated by vertical lines. The Reg_Prop repeats are colored pink, the Asp-box repeats are light blue and residues not belonging to any of these motifs are grey. The structural model is in cartoon representation with the side chain of the tryptophan in the 10^th ^position of the Asp-box (W111) shown in sticks. It is based on 2cn2 and is probably rather inaccurate in most respects (sequence id. 15%), but it illustrates well how the two motifs are likely to be arranged in special hybrid propeller blades. The fourth strand is missing in the modeled blade, which may appear to indicate that the model is erroneous in this area, but a lack of the fourth strand is indeed quite commonly observed for Asp-box β-propeller blades.

Among the yet uncharacterized putative Asp-box β-propellers a heterogeneous group of putative hybrid β-propellers deserves specific mentioning. Hybrid β-propeller domains containing more than one type of propeller-associated repeats are rare in known structures, yet Asp-boxes are found in between other propeller-associated repeats in several sequences. Examples are a putative archeal kelch hybrid (InterPro accession A3CWT3) and some putative bacterial PQQ (e.g. Q3KK02) and Reg_Prop (e.g. A9KRI6) hybrids. The Reg_prop motif is related to the WD40 and PQQ repeats and is according to Pfam believed to promote a β-propeller fold, although this has not yet been shown. Interestingly, it seems that in some cases, Asp-boxes are even found in the same blades as other propeller-associated repeats. The positioning of the Asp-box and Reg_prop motifs in the sequence of the YP_001558799.1 hypothetical protein from *Clostridium phytofermentans *(A9KRI6) suggests the presence of several hybrid blades as the two motifs follow each other in immediate or almost immediate succession with often just three amino acids in between (Figure 4). Such hybrid-blade β-propellers have to our knowledge neither been described nor predicted before. A recombination event involving just two strands does not seem particularly likely and the blades of such propellers may thus represent ancestral units that have since diversified into blades retaining just one or the other repeat. Alternatively, they may reflect that some propeller-associated repeats have evolved more than once e.g. the Reg_prop motif may in one case have evolved in a blade already containing an Asp-box.

### Structural alignment of Asp-box β-propeller blades

**Figure 5:**
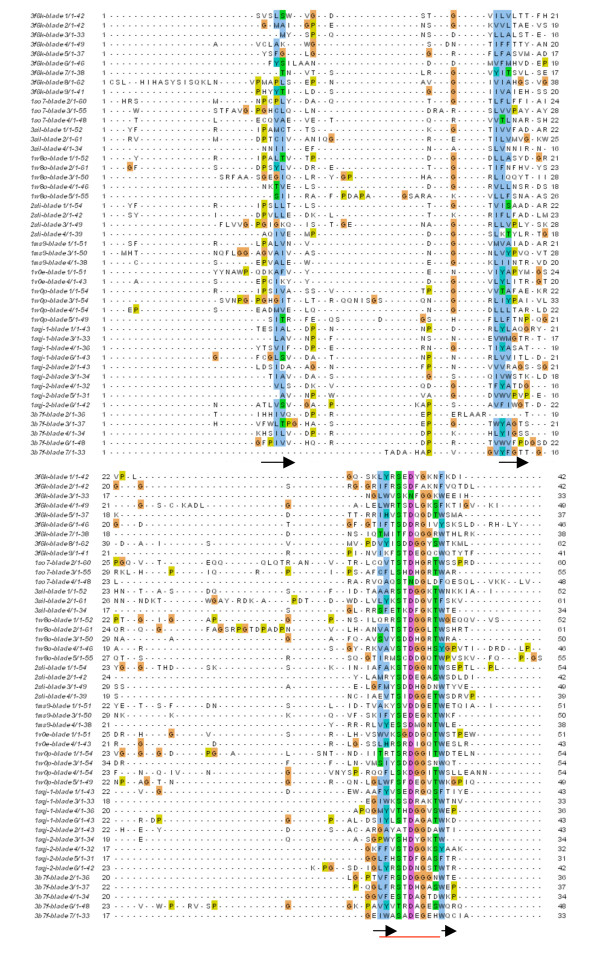
**Multiple structural alignment of β-propeller blades containing Asp-box repeats**. This alignment was made using all blades with an Asp-box from ten representative structures: Sortilin from man (3f6k), cytoplasmic sialidase Neu2 from man (1so7), sialidase from *S. typhimurium *(3sil), sialidase from *M. viridifaciens *(1w8o), intramolecular trans-sialidase from the leech *M. decora *(2sli), trans-sialidase from the trypasnosome parasite *T. cruzi *(1ms9), endosialidase from bacteriophage K1F (1v0e), sialidase from *V. cholera *(1w0p), cellobiohydrolase from the fungus *Geotrichum sp. M128 *(1sqj; 1sqj-1 and 1sqj-2 refer to the first and second propeller respectively), and YP_299179.1 from *Ralstonia eutropha *(3b7f). Numberings are according to blade positions i.e. position 1 is the first position in the given blade rather than in the protein in which it occurs. The color scheme is Clustal X. The position of the Asp-box is marked by a red line, and in order to give an impression of the general localization of strands, black arrows representing the four strands of the sixth blade of 3b7f are shown beneath the alignment.

**Figure 6:**
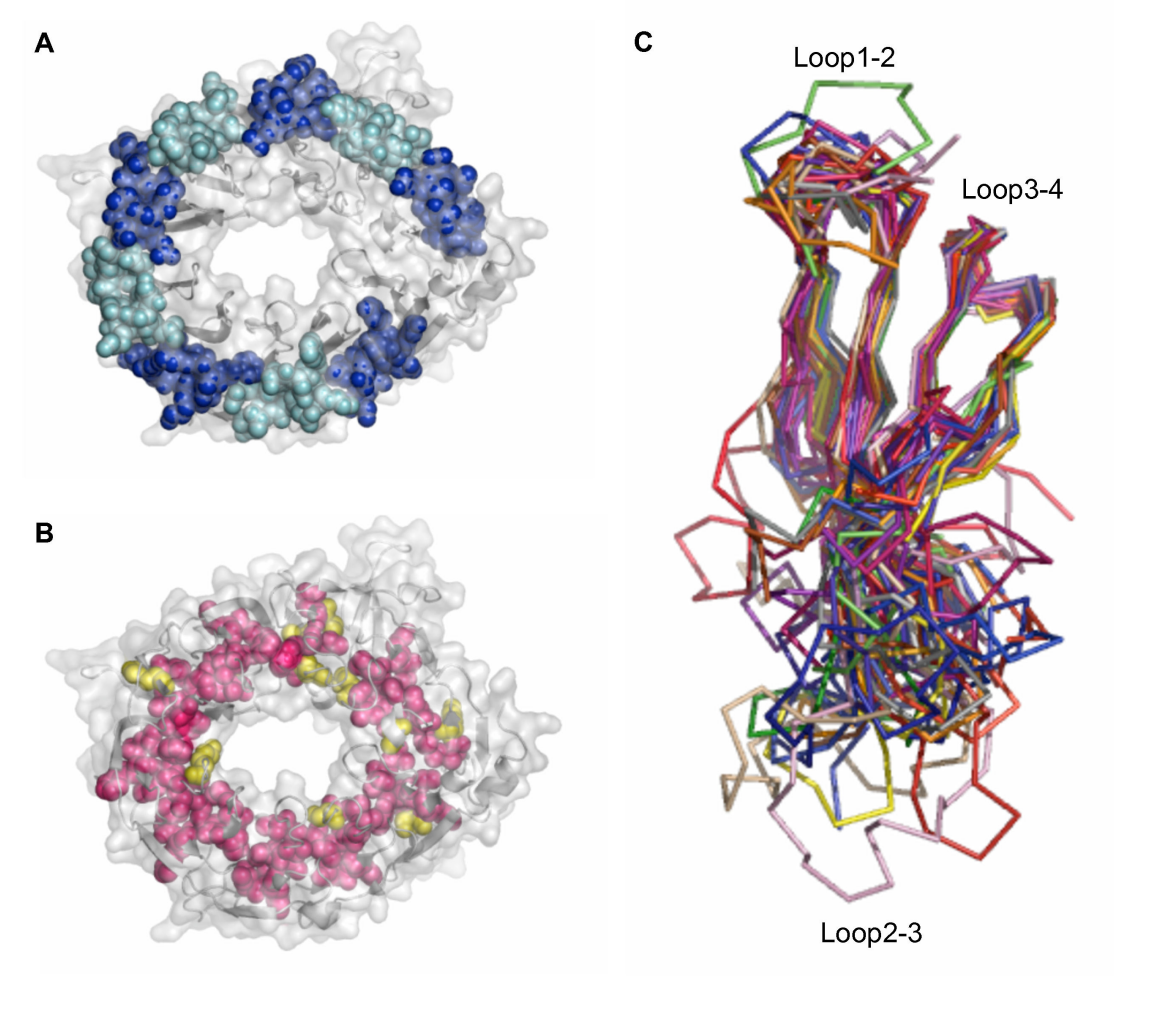
**Common structural features of Asp-box β-propellers and overlay of the β-propeller blades**. A. The Asp-box β-propeller of sortilin is shown in grey cartoon with a semi-transparent surface. The spheres represent the Asp-boxes, which are colored alternately blue (uneven blades) and cyan (even blades). B. Same as in (A) except that the spheres shown here represent the conserved hydrophobic positions instead of the Asp-boxes. Residues in these positions that are indeed hydrophobic in human sortilin are colored pink and polar/charged residues are colored yellow. C. Multicolored ribbon overlay resulting from the multiple structural alignment shown in Figure 5.

A multiple structural alignment was made of all blades containing an Asp-box from ten representative Asp-box β-propellers (Figure 5). The Asp-box itself is clearly the most well-conserved feature of these blades, but there are also several conserved hydrophobic positions; a doublet in strand 1, a triplet in the beginning of strand 2 and a doublet in strand 3. These residues and some surrounding residues in the first three strands are furthermore characterized by having a strong propensity for forming β-strands. Conversely, there is in between the strands, a high incidence of residues with strong propensity for forming turn structure i.e. G, P, S, D and N [32]. The conserved hydrophobic positions overlaps a set of positions previously identified as being generally conserved in β-propellers [23] and the observed conservation of hydrophobic positions and the pattern of strand and loop propensities do therefore not appear to represent specific properties of the Asp-box family, but rather of β-propellers in general. It is thus not surprising that a structural alignment including all blades of the same set of proteins reveals that the hydrophobic positions are conserved in all the blades, regardless if they contain an Asp-box or not (see additional file 2: Suppl_Figure1.pdf for figure). It may however be noted that these patterns can probably be readily recognized by fold recognition programs that employ secondary structure algorithms and sensitive sequence profile matching, and thus be useful for evaluating if an uncharacterized Asp-box repeat protein adopts a β-propeller fold or like reelin adopts an alternative fold. Mapping out the conserved residues in the structures clearly reveals the likely reasons for why they are conserved; the hydrophobic residues basically make up the hydrophobic core, and as previously described the Asp-boxes mediate stabilizing blade to blade interactions at the outer rim (Figure 6A). The overlay resulting from the structural alignment reveals that the two ends of the blades and thereby also the top and bottom faces of the propeller i.e. the faces that comprise the N-terminal and C-terminal parts of the inner strands respectively, have very distinct properties. The end comprising loop1–2 (the loop between strand 1 and 2) and loop3–4 is very well-defined, whereas the opposite end comprising loop2–3 is extremely variable (Figure 6B). The strands generally overlay fairly well although with some exceptions, but there is a marked variation in lengths, the outer strands are sometimes reduced to loop structure and the inner strands may encompass various insertions, e.g. a signature of the GH33 family is a β-bulge in the inner strand of blade 3, which we find to conform to the consensus sequence G-X-G-X-G. It is these features that underlie the previously noted distinctive irregularity of members of the Asp-box β-propeller family as compared to other β-propellers [23]. The conserved positions of the Asp-box are restricted to just one loop, whereas they are scattered over longer regions in most other propeller-associated repeats. This probably leaves more room for structural variation in Asp-box β-propellers than in other families and could thus at least partly explain why they contain more irregularities. Interestingly, the set of residues involved in ligand binding and catalysis in all known Asp-box glycosidases are located in the variable loop2–3 on the top face of the propeller. In sortilin it appears that ligand binding generally occurs in the tunnel rather than at the loops of one of the two faces [19], but two hydrophobic loops that strongly protrude from the structure are expected to be of functional significance, and these loops are indeed found on the top face. It thus seems, that the variable top face is generally used for functional activities, whereas the well-defined bottom face comprising the Asp-boxes is generally used for supporting the structural integrity of the fold. In new structures of Asp-box β-propellers, we therefore recommend that focus should be on the top face in any attempts to identify functionally important residues. It is beyond the scope of this paper to systematically investigate if this structural and functional distinction between the two faces also applies to other families of β-propellers, but it should be mentioned that most β-propellers bind ligands at the top face [44], although binding can also occur at the bottom face [45] or at the side of the domain [46,47]. Phasing by molecular replacement can be challenging for Asp-box β-propellers e.g. phases for the *M. viridifaciens *GH33 sialidase could not be obtained using the GH33 sialidase structures from *V. Cholera *or *S. typhimurium *as search models [5]. There are now so many known structures of GH33 proteins, that phasing by molecular replacement is a routine matter for members of this family, but Asp-box β-propellers that are slightly more distantly related to potential templates, can still prove challenging. For such cases, we recommend that the search models should include the strands and Asp-boxes, whereas loop2–3 should be removed completely from all blades. Furthermore, the side chains of the conserved Asp-box residues should not be trimmed. Finally we suggest that theoretical models resulting from homology modeling or fold recognition should be interpreted with great caution, since the functionally interesting parts are likely to be on the variable top face, which will be very challenging to model accurately, unless a very closely related template is used.

### The 10-bladed β-propeller fold

The Asp-box β-propeller of the recently published structure of sortilin deserves special attention in being the first and so far only observed 10-bladed β-propeller [19]. As a measure for the impact on packing geometry of an increased number of blades, we have measured the strand to strand distances and tunnel dimensions of selected examples of 6-, 7- and 10-bladed Asp-box β-propellers (Table 3). The distances between the second or third strands remain almost constant regardless of the number of blades, but the distances between the inner first strands or outer fourth strands increase and decrease respectively when the number of blades increases. Thus, as more blades are accommodated they tweak relative to each other, so that the strand to strand distances become more even for all four strands. It is well known that residues of the inner strands of β-propellers pack by intercalation of hydrophobic residues whereas residues in the other strands pack in more variable ways [23,48], but in the 10-bladed fold the inner strands have moved so far away from each other that they now also pack in more variable ways, much like the middle strands of the smaller β-propellers. It has previously been predicted that the tunnel of a 10-bladed β-propeller would have a diameter of approximately 25 Å [23], but we find it to be considerably larger, roughly 25 by 37 Å in the equatorial plane and expanding drastically in size when moving towards the top face. Thus, the unforeseen increase in inner strand distances results in the tunnel of sortilin being unexpectedly large, which is crucial for its mode of ligand binding [19]. The shape of the tunnel is quite variable e.g. in sialidases it narrows towards the top face as in most other β-propellers [23], whereas in sortilin it narrows towards the bottom face. However, the tunnel of the 5-bladed β-propeller of tachylectin-2, which is however not an Asp-box protein, likewise narrows towards the bottom face, suggesting that the shape of the tunnel is not dictated by the number of blades in the propeller. The reason why 10-bladed β-propellers are rare is not quite clear and it is difficult to assess if the Asp-box family is better suited than other β-propeller families for adopting a 10-bladed fold. A characteristic of the Asp-box is however, that all conserved positions except the conserved aromatic residue are used solely to support the structure of the Asp-box itself, so that the positions generally used for interacting with other blades are free to vary and thus adapt to different structural contexts. It is however possible that the 10-bladed fold is indeed not exclusive to the Asp-box family. Some members of the RCC1 superfamily thus have ten highly conserved RCC1-like repeats that likely form a 10-bladed β-propeller, although it can not be precluded that they rather form a tandem of e.g. two 5-bladed β-propellers [49]. Furthermore, engineered proteins with nine or ten WD40 repeats have been expressed, but it remains to be shown that they actually fold as 9-bladed or 10-bladed β-propellers [50].

**Table 3:** Metrics of Asp-box β-propellers as function of the number of blades

Distances (Å)	6-bladed, 1v0e	7-bladed, 3bf7	10-bladed, 3f6k
Strand 4-4	20	19	17

Strand 3-3	15	15	15

Strand 2-2	11	11	12

Strand 1-1	5	7	10

Tunnel dimensions	Occluded	9 by 11	25 by 37

Strand to strand distances and tunnel dimensions are shown for selected examples of Asp-box β-propellers with different sizes; the 6-bladed β-propeller of K1F bacteriophage sialidase (1v0e), the 7-bladed β-propeller of YP_299179.1 (3bf7) and the 10-bladed β-propeller of sortilin (3f6k).

## Conclusion

Here we have examined the occurrence of the Asp-box and investigated its structure and structural contexts in detail and we also provide the first thorough overview, classification and detailed structural analysis of Asp-box β-propellers. In addition we have dealt with the current confusion regarding the functions of Asp-box repeats, which is an important point to clarify given their wide distribution and occurrence in physiologically and medically important protein families. Finally, we have analyzed the packing principles of the 10-bladed β-propeller fold, which has so far exclusively been observed in the Asp-box β-propeller family. The Asp-box and the shorter variant, the s-Asp-box, both occur in different folds, but are most prominently found in β-propellers. They can be viewed as micro-scaffolds where the conserved residues ensure a stable very well-defined fold of the motifs and the residues in variable positions are free to carry out other functions. Generally, the Asp-box has a structural role both when it occurs as singlet and when it occurs as repeats, but it can also be used for other purposes i.e. it is involved in catalysis and nucleotide binding in microbial ribonucleases. In β-propellers, the Asp-box repeats support structural integrity by mediating blade to blade interactions, but although they may have additional roles in some cases, a survey of the current evidence does not support the previously suggested functions as secretion signals or carbohydrate binding motifs. Putative Asp-box β-propellers occur in all major branches of life and appear to constitute one of the most common β-propeller families in bacteria. Furthermore, Asp-box repeats occur together with a long list of different domains, suggesting a much wider functional range of the Asp-box β-propeller family than has hitherto been acknowledged. Most or all Asp-box and s-Asp-box β-propellers probably share a common ancestor, which would place the GH32, GH33, GH43, GH58, GH74 and Vps10-D families in the same superfamily. However, since the Asp-box and s-Asp-box are quite short motifs and occur in β-sheets with different topologies, it is certainly possible that they in some cases have evolved by convergence, e.g. it seems likely that the s-Asp-box in the Ribonuclease H-like fold of *Thermotoga maritima *type III PanK has evolved by recent chance convergence, as it does not generally occur in other related proteins with this fold. The discovery of novel putative hybrid-blade β-propellers is intriguing and may prompt a revised view of β-propeller evolution, but detailed evolutionary analysis should await experimental validation of the predicted fold. A multiple structural alignment of representative Asp-box β-propeller blades discloses that not only the Asp-boxes are conserved, but also a set of hydrophobic positions with high propensity for β-strand formation. These appear to be common to all β-propellers and may be helpful in predicting if an uncharacterized Asp-box repeat protein adopts a β-propeller fold. The structural alignment furthermore shows that the two faces of Asp-box β-propellers are strikingly different and further examination reveals that they are indeed also used for different purposes; the top face is extremely variable and is used for functional purposes whereas the bottom face displays limited variability and supports the fold. This finding will likely be very useful for interpretation of future structures and can also, along with the classification into organizational types, be used for aiding the making and evaluation of theoretical models. The analysis of the 10-bladed fold, which has so far only been observed in the Asp-box sequence family of β-propellers, reveals that accommodation of more blades in an Asp-box β-propeller, and indeed probably in any β-propeller, is accompanied by tweaking of the blades, so that the distances between inner strands becomes longer and those of the outer strands become shorter. This increase in inner strand distances explains the unexpectedly large size of the ligand binding tunnel of sortilin, which is crucial for the function and regulation of this receptor [19] and probably of the Vps10-D receptors in general.

## Methods

### Classification and sequence analysis

The nomenclature for glycosidase families that encompass Asp-box β-propeller domains was looked up in the CAZy database for sequence-based classification of carbohydrate active enzymes [51]. The Asp-box consensus sequence was determined from the full alignment of the BNR entry in the Pfam release 22.0 database [52] and Pfam was also used to identify the various motifs and domains that can be present in proteins with at least two Asp-box repeats. It should be noted however that Pfam release 23.0 contains a much smaller set of sequences in the BNR entry. This is also reflected in the current version of interpro (20.0) as it is based in part on Pfam. Information on the phylogenetic distribution of Asp-box repeats and other propeller-associated repeats was retrieved from InterPro 18.0 [53], putative hybrid propellers were identified using both Pfam and InterPro, and fold recognition of YP_001558799.1 was carried out with the Phyre server [54].

### Structure-based search for Asp-box proteins

The structural search for Asp-box motifs was carried out with SPASM [55] using the first Asp-box of 2bf6 (residues 302–311) and the July_08 pdb database [56] as inputs. The key search criteria were as follows; max superpositioning RMSD was set to 1.0 Å, max Cα-Cα distance mismatch was set to 1.5 Å, BLOSUM 45 was used as substitution matrix, the directionality and gaps of the peptide trace was conserved and only the Cα atoms were used for centre-of-gravity. The search for the one residue shorter motif, which we call the s-Asp-box, was also carried out using SPASM. Here the conserved S, D and W of the first s-Asp-box of 1y4w (S75, D77, W81) was used as input. The key search criteria were as follows; max superpositioning RMSD was set to 0.7 Å, max Cα-Cα distance mismatch was set to 0.5 Å, max side chain to side chain distance mismatch was set to 2.0 Å, the only allowed substitutions were S75/T, D77/N and W81/F/Y, the directionality and gaps were conserved, and here both the Cα and side chain atoms were used for centre-of-gravity. For both search procedures, the sequences of the hits were checked and representative hits were manually inspected in PyMol [57] in order to avoid potential false negatives. Importantly, SPASM proved efficient in finding the Asp-boxes and s-Asp-boxes of proteins that were already known to contain these motifs, demonstrating the robustness of the approach.

### Structural alignment and analysis

Borders for individual Asp-box β-propeller blades (first residue in the first strand to last residue in the fourth strand) were identified using PDBsum [58]. The multiple structural alignments of blades and individual Asp-box/s-Asp-box motifs were then made with the MUSTANG program [59] and the results were presented using PyMol and the JalView alignment editor [60]. Information on hairpin, turn types and phi and psi angles for Asp-boxes and s-Asp-boxes was retrieved from PDBsum. Strand to strand distances were measured between equatorial Cα atoms in PyMol and averaged over all blades in the analyzed structures. The dimensions of the β-propeller tunnels were measured between any surface-exposed atoms in the equatorial plane.

## Authors' contributions

EQ conceived the project, carried out the computational and structural analysis of the Asp-box and the Asp-box β-propeller family and wrote the manuscript. ST participated in the structural analysis of the Asp-box, in the design of the project and in writing the manuscript. Both authors read and approved the final manuscript

## Supplementary Material

Additional file 1 **Alignment of blades of propellers**. Sequence alignment. Click here for file

Additional file 2 **Domains and motifs that co-occur with Asp-box repeats**. List of domains co-occuring with Asp-box. Click here for file
