# Supplementary material for: Sequence and structural analysis of the Asp-box motif and Asp-box beta-propellers; a widespread propeller-type characteristic of the Vps10 domain family and several glycoside hydrolase families
Source: BMC Struct Biol. 2009 Jul 13;9:46. doi: 10.1186/1472-6807-9-46 (PMC2716378; doi:10.1186/1472-6807-9-46)
Supplement: Additional file 1 — Alignment of blades of propellers. Sequence alignment. [file 1472-6807-9-46-S1.pdf]

Adeno_shaft, ADH_N, ADH_zinc_N, Ank, Autotransporter, Bac_rhamnosid, cadherin, Calx-beta, CARDB, CBM_1, CBM_2, CBM_3, Chlam_PMP, Clathrin_propel, Cohesin, Collagen, Collar, Dockerin_1, CybS, DUF11, DUF291, DUF386, DUF789, EGF, EGF_2, F5_F8_type_C (CBM32), FG-GAP, Fil-haemagg, FIVAR, Flagellin_N, Flg_new, fn3, GLUG, HaemolysinCabind, He-PIG, HAMP, IU_nuc_hydro, Laminin_G_2, kelch_1, kelch_2, LDL_recept_a, LDL_recept_b, LectinC, Lectin_legB, LysM, MAM, Metallophos, PA, PD40, Pentapeptide, Peptidase_S8, Phage_T7, phage_tail_N, PKD, RCC1, pro_isomerase, PT repeat, Reeler, Reg_prop, RHS_repeat, Ribonuclease_BN, Ricin_B_lectin, RPE65, Sialidase, Sial-lect-inser, SpoIIE, Staphyllocoagulase, TIG, VWA, W_rich_C, YSIRK, Y_Y_Y

Supplementary box 1. Domains and motifs that co-occur with Asp-box repeats

The domains and motifs that can co-occur with Asp-box repeats are named here using Pfam nomenclature and listed in alphabetical order. To aid interpretation we have color coded the domains by a crude division into functional categories; protein-protein interaction –red, cell surface attachment –blue, carbohydrate binding –green, hydrolysis –magenta, and other, unknown or multiple functions –black.
