# Supplementary material for: Sequence and structural analysis of the Asp-box motif and Asp-box beta-propellers; a widespread propeller-type characteristic of the Vps10 domain family and several glycoside hydrolase families
Source: BMC Struct Biol. 2009 Jul 13;9:46. doi: 10.1186/1472-6807-9-46 (PMC2716378; doi:10.1186/1472-6807-9-46)
Supplement: Additional file 2 — Domains and motifs that co-occur with Asp-box repeats. List of domains co-occuring with Asp-box. [file 1472-6807-9-46-S2.pdf]

Supplementary figure 1. Multiple structural alignment of all blades from selected Asp-box β-propellers.

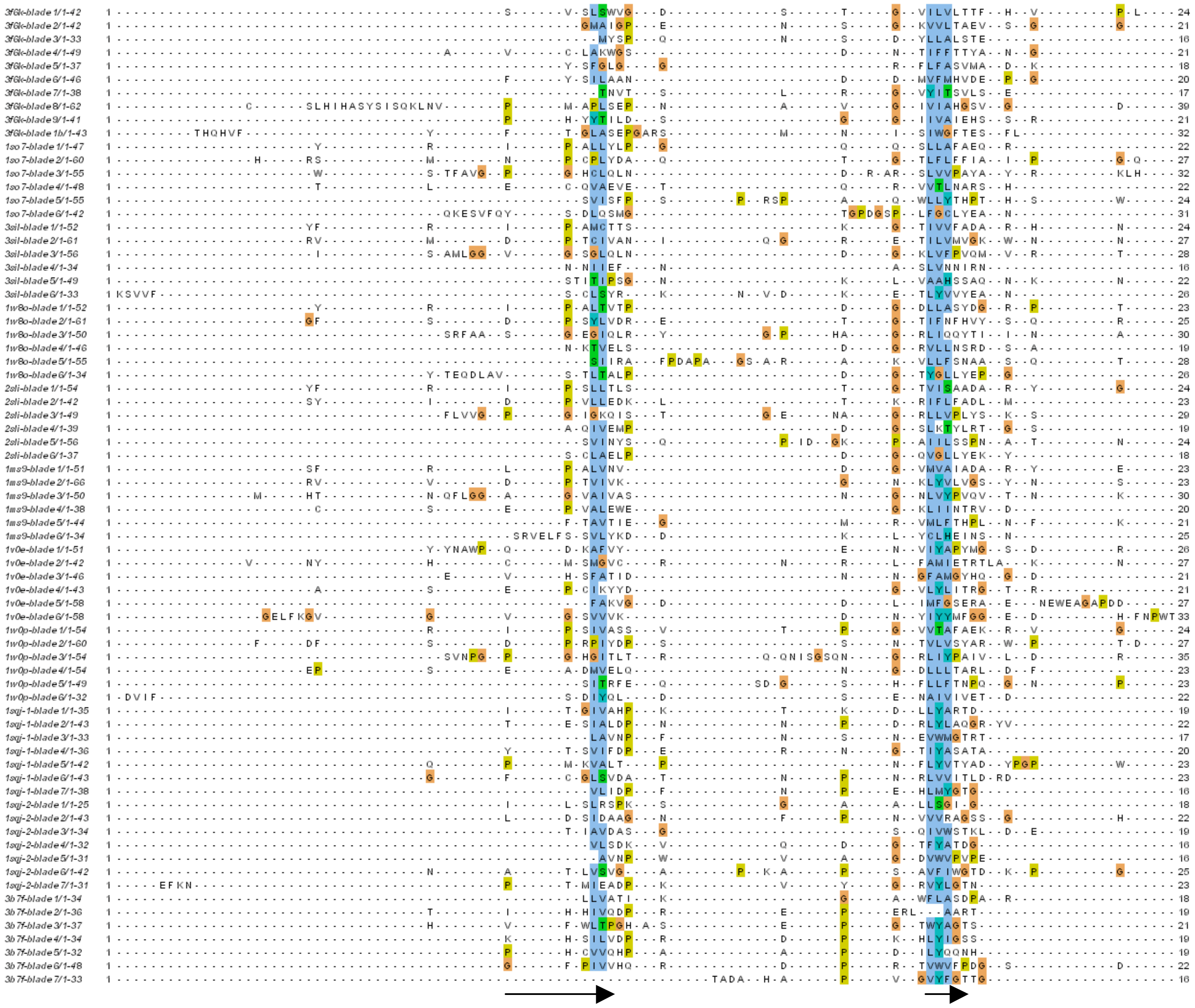

Supplementary figure 1 (part 1)

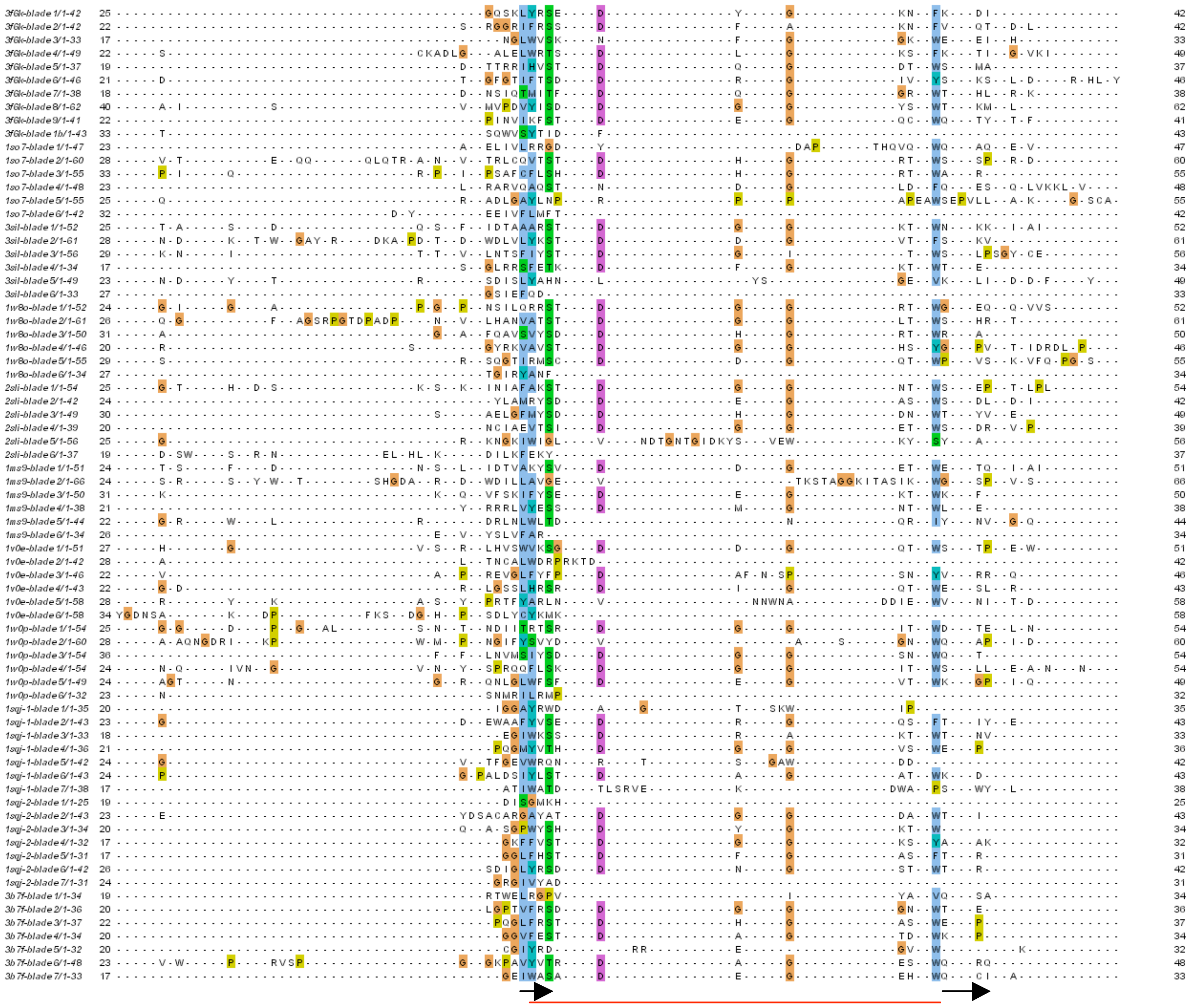

Supplementary figure 1 (part 2)

Supplementary figure 1. Multiple structural alignment of all blades form selected Asp-box β-propellers. This alignment includes propeller blade sequences extracted from the same protein sequences as used for the alignment shown in figure 5, but here the blades that do not contain an Asp-box, were also included. As in Figure 5, the numberings are according to blade positions i.e. position 1 is the first position in the given blade rather than in the protein in which it occurs. The position of the Asp-box is marked by a red line and the four strands of the sixth blade of 3b7f blade are represented by black arrows shown beneath the alignment. The color scheme is Clustal X.
